# Joint effects of individual socioeconomic status and residential neighborhood context on vaginal microbiome composition

**DOI:** 10.3389/fpubh.2023.1029741

**Published:** 2023-01-24

**Authors:** Meredith Dixon, Anne L. Dunlop, Elizabeth J. Corwin, Michael R. Kramer

**Affiliations:** ^1^Department of Epidemiology, Rollins School of Public Health, Emory University, Atlanta, GA, United States; ^2^Department of Gynecology and Obstetrics, Emory University School of Medicine, Atlanta, GA, United States; ^3^Columbia University School of Nursing, New York, NY, United States

**Keywords:** microbiota, social environment, neighborhood characteristics, pregnancy, United States, dysbiosis

## Abstract

**Introduction:**

The vaginal microbiome is a dynamic ecosystem that is important for women's health. Its composition has been associated with risk for menopausal symptoms, sexually transmitted infections, gynecologic cancer, and preterm birth. Conventional risk factors for a vaginal microbiome linked with these adverse health outcomes include sexual behaviors, hygiene practices, individual social factors, and stress levels. However, there has been limited research on socio-contextual determinants, and whether neighborhood context modifies the association with individual socioeconomic factors.

**Methods:**

Socioeconomically diverse pregnant African American women in Atlanta, Georgia (*n* = 439) provided residential addresses and first trimester vaginal swab samples, which underwent sequencing, taxonomic classification, and assignment into mutually exclusive CST (community state types) *via* hierarchical clustering. Linear probability models were used to estimate prevalence differences (PD) for the associations of neighborhood factors with vaginal microbiome CST and to evaluate for additive interaction with maternal level of education, health insurance type, and recruitment hospital.

**Results:**

Factors such as higher (vs. lower) maternal education, private (vs. public) insurance, and private (vs. public) hospital were associated with higher prevalence of *Lactobacillus*-dominant vaginal microbiome CSTs typically associated with better health outcomes. When considering the joint effects of these individual socioeconomic status and residential neighborhood factors on vaginal microbiome CST, most combinations showed a greater than additive effect among the doubly exposed; however, in the case of local income homogeneity and local racial homogeneity, there was evidence of a crossover effect between those with less-advantaged individual socioeconomic status and those with more-advantaged individual socioeconomic status. Compared to women at the public hospital who lived in economically diverse neighborhoods, women at the private hospital who lived in economically diverse neighborhoods had a 21.9% higher prevalence of *Lactobacillus*-dominant CSTs, while women at the private hospital who lived in less economically diverse neighborhoods (the doubly exposed) had only an 11.7% higher prevalence of *Lactobacillus*-dominant CSTs, showing a crossover effect (interaction term *p*-value = 0.004).

**Discussion:**

In this study, aspects of residential neighborhood context were experienced differently by women on the basis of their individual resources, and the joint effects of these exposures on vaginal microbiome CST showed a departure from simple additivity for some factors.

## Introduction

The vaginal microbiome is a dynamic ecosystem that is important for women's health. Most of the indigenous microorganisms in the vaginal environment exist in harmony with their host and play an essential defensive role against harmful invading pathogens (1, 2). Disruptions in the mutualistic relationships occurring in the vaginal microbiome have been linked to increased risk for menopausal symptoms (3), sexually transmitted infections (4), gynecologic cancer (5), bacterial vaginosis (6, 7), and preterm birth (8, 9).

The vaginal microbiome consists of a multitude of microorganisms, including bacteria, viruses, and fungi. In research studies, the composition of the vaginal microbiome is commonly conceptualized by community state type (CST) assignment, defined by dominant species in the vaginal microbial community. One of the predominant bacteria in a healthy microbiome is *Lactobacillus*, which aids in maintaining the desired acidic environment that wards off harmful pathogens. Although most *Lactobacillus* species are beneficial, microbiota dominated by *Lactobacillus iners* are not as strong at inhibiting the growth of anaerobes as microbiota dominated by the other species of *Lactobacillus*, but the role of *L. iners* in vaginal health is still unclear (10). Additionally, some vaginal microbiomes are dominated by anaerobic bacteria, and the lack of *Lactobacillus* species in these environments does not create the low pH environment typically characteristic of vaginal health (2, 8, 11–13).

Factors that tend to affect levels of *Lactobacillus* in the vaginal microbiome and subsequent eubiosis or dysbiosis include: transmission of microorganisms through maternal inoculum, sexual activity, and cohabitation; host factors such as genetics and hormone levels; and behavioral factors such as substance use, diet, vaginal hygiene practices, antibiotic use, and hormonal contraceptive use (14–16). The vaginal microbiome composition is also dependent on age, and women can transition between CST states over their lifespans (2, 8). Additionally, studies have found that distribution of vaginal microbiome patterns vary by race and ethnicity and that there are “an appreciable percentage of asymptomatic and healthy women harboring an array of diversified strictly and facultative anaerobic microbes” (2).

There is some evidence that vaginal microbiome composition varies by individual levels of psychosocial stress and other social factors (17–21), and there is increasing awareness that these factors are not purely a result of individual choices or behaviors, but are also represented by differences in experienced social context, including the residential neighborhood context (22, 23). It is therefore plausible that where someone lives could affect the health of the vaginal microbiome. For example, neighborhood measures of the material economic environment might be related to access to resources, which could in turn possibly affect diet and other behaviors associated with microbiome composition (24). Similarly, neighborhood measures of social capital might reflect the social network, which could subsequently impact social support, norms and beliefs around hygiene practices, and sexual activity (25–27). Likewise, neighborhood measures of stressful or violent environments might be correlated with psychosocial health, which could also influence microbiome composition (28). Comparably, neighborhood measures of inequality and diversity might correspond to social hierarchy and power structures, which could affect both access to resources and individual stress levels (29).

Additionally, it is plausible that aspects of social and neighborhood context could be experienced differently on the basis of individual resources and experiences. Therefore, this study aims to explore the joint effects of individual socioeconomic status and neighborhood residential context on the composition of the vaginal microbiome. Without much literature to date on social risk factors for microbiome dysbiosis and subsequent disease, this work has the potential to provide novel insight into these associations and to help generate focused hypotheses for future research in this field.

## Methods

The Emory University African American Microbiome in Pregnancy Cohort Study recruited women who presented with singleton pregnancies for their first prenatal visits between 8 and 14 weeks' gestation, who self-identified as African American, who were between 18 and 40 years of age, who could comprehend written and spoken English, and who were experiencing no chronic medical conditions (30). Two hospitals, both in Atlanta, Georgia, were sampled by design in order to achieve socioeconomic diversity. Emory University Hospital Midtown is a private hospital and Grady Memorial Hospital is a publicly-supported safety net hospital; both hospitals are staffed by Emory University Obstetric & Gynecological physicians. Vaginal swab samples and questionnaire data were collected at both the initial visit and at a subsequent prenatal care visit between 24 and 30 weeks' gestation. The vaginal swab samples then underwent sequencing of the 16S rRNA gene (V3–V4 region), taxonomic classification, and assignment into mutually exclusive community state types using hierarchical clustering. These data sequencing procedures have been described in detail previously (8, 30), but briefly are as follows: DNA was extracted from participant swab samples using the DNeasy PowerSoil Kit (cat# 12888-100, Qiagen), followed by DNA quantification using a threshold of 5 ug/nL to identify samples identified as borderline in terms of DNA yield; for cases identified as borderline, DNA quality was assessed on a 2% agarose gel and quantitated with the Broad Range Quant-It kit from ThermoFisher Scientific (Q33130). Samples from participants that included DNA visible on the gel were sequenced as were 30 no-template controls (containing all assay components except for DNA to verify lack of contamination across reagents and samples) and positive controls (a mixture of 20 vaginal specimens of known composition). Microbial composition was characterized by DNA sequencing of the 16S rRNA gene. Amplification of the V3–V4 regions of the 16S rRNA gene was performed using a two step-PCR as described previously (31). Briefly, the first PCR used the short 16S rRNA gene specific primers 319F (ACACTGACGACATGGTTCTACA[0–7]ACTCCTRCGGGAGGCAGCAG) and 806R (TACGGTAGCAGAGACTTGGTCT[0–7]GGACTACHVGGGTWTCTAAT) where the underlined sequence is the Illumina sequencing primer sequence and [0–7] indicate the presence of an heterogeneous pad sequence to improve sequencing quality (32), for a total of 20 cycles. The second step extends the amplicon with the Illumina required adaptor sequences and the sample specific dual barcode system *via* 10 cycles with primers H1 (AATGATACGGCGACCACCGAGATCTACACNNNNNNNNACACTGACGACATGGTTCTACA) and H2 (CAAGCAGAAGAC GGCATACGAGATNNNNNNNNTACGG TAGCAGAGACTTGGTCT) where NNNNNN identifies a sample specific barcode sequence and the underlined sequence corresponds to the Illumina sequencing primer for priming to the first step amplicon (32). Amplicons were visualized on a 2% agarose gel, quantified, pooled in equimolar concentration, and purified prior to sequencing on an Illumina HiSeq 2500 (San Diego, CA, USA) modified to generate 300 bp paired-end reads (33). Extraction and PCR negative controls as well as a positive control composed of a mixture of 20 vaginal biological specimens of known composition were processed in parallel. Also as described previously (8), the sequences were de-multiplexed using the dual-barcode strategy, that utilizes a mapping file to link barcode to samples and split_libraries.py, a QIIME-dependent script (34). The resulting forward and reverse fastq files were split by sample using the QIIME-dependent script split_sequence_file_on_sample_ids.py, and primer sequences were removed using TagCleaner (version 0.16) (35). Further processing followed the DADA2 Workflow for Big Data and dada2 (v. 1.5.2) (https://benjjneb.github.io/dada2/bigdata.html) (36). Forward and reverse reads were trimmed using lengths of 255 and 225 bp, respectively, and were filtered to contain no ambiguous bases, and to have a minimum quality score of 2; they were also required to contain less than two expected errors based on their quality score. The relationship between quality scores and error rates were estimated for both sequencing runs to reduce batch effects arising from run-to-run variability. Reads were assembled and chimeras removed as per dada2 protocol. From the 439 samples, reads were grouped into 666 amplicon sequence variants (ASVs). After removing taxa that were only identified to the family level or higher, we retained 19,571,748 reads in 601 ASVs. After the glomming and trimming operation, the data remaining comprised 19,544,087 reads in 324 taxa (with a range of 2,978 to 181,072 reads per sample).

Assignment into mutually exclusive community state types using hierarchical clustering was as follows: CST I being predominated by *L. crispatus*, CST II by *L. gasseri*, CST III by *L. iners*, CST IV being defined as lacking *Lactobacillus* predominance and comprising a diverse set of strict and facultative anaerobes, and CST V being predominated by *L. jensenii* (8). Participants in the present study include the first consecutively enrolled 439 women for whom at least one vaginal swab sample from the enrollment study visit was available for DNA extraction and gene sequencing.

The individual exposure measures investigated in this study represent three aspects of socioeconomic status and resource accessibility: maternal education level, maternal insurance type, and maternal recruitment hospital. Education level was self-reported at the initial visit as one of the following: less than high school, high school graduate or GED, some college, or college graduate. For the present study, maternal education level was dichotomized as either graduating from college or having less than a college degree. We theorized that the differences between these two groups might be the most substantial, and we confirmed this hypothesis with our data, along with verifying that our results did not meaningfully change when using a dichotomous version of this variable instead of the multi-categorical version of it. Maternal insurance type was initially classified as either private insurance, low income Medicaid, or Right from the Start (RSM) Medicaid, which is available to pregnant women even if they do not qualify for low income Medicaid. For the present study, maternal insurance type was dichotomized as either having private insurance or not having private insurance. We theorized that the differences between these two groups might be the most substantial, and we confirmed this hypothesis with our data, along with verifying that our results did not meaningfully change when using the dichotomous version of this variable instead of the multi-categorical version of it. Maternal recruitment hospital was classified as either Emory Hospital or Grady Hospital, based on where the participants were receiving their prenatal care. For the remainder of the paper, Emory will be referred to as the private hospital, and Grady will be referred to as the public hospital.

The neighborhood exposure measures investigated in this study were: neighborhood deprivation index, percentage of households that moved in the last year, census response rate, violent crime rate, local income homogeneity, and local racial homogeneity. These exposures were assigned to individual participants based on self-reported maternal residential addresses, which were geocoded into census tract assignments. The neighborhood deprivation index was used as a measure of the residential material and economic environment and was a composite variable obtained as the first principle component (weighted average) of the following eight variables (all obtained from the American Community Survey), as described by Messer et. al. (37): (i) percent of males in management, (ii) percent of households with greater than one person per room, (iii) percent of individuals with income below the federal poverty level, (iv) percent of families with female headed households with dependent children, (v) percent of households with annual income <$30,000, (vi) percent of households with public assistance income, (vii) percent of adults unemployed, and (viii) percent of adults with no high school education. The percentage of households that moved in the last year and the census response rate were used as measures of residential stability and social capital; the former was retrieved from the American Community Survey, and the latter was obtained from the Census Bureau as a percent of housing units that completed the 2020 census. The violent crime rate at the census tract level was extracted from Environmental Systems Research Institute (ESRI) modeled estimates as a measure of stressful environments (38).

Finally, we estimated measures of local income homogeneity and local racial homogeneity derived from cellphone activity space of non-residents visiting the participant's neighborhood. Conventional approaches to measuring residential environment rely on indicators from the American Community Survey describing attributes of the population who live in the neighborhood. However, the social experience of a place is likely not defined solely by the people who sleep there (e.g., residents) but also by the experiences and exposures that result from routine mobility during the day. To quantify aspects of the racial and economic diversity of each woman's neighborhood during the day (e.g., from non-residents visiting), we used a large database of daily mobility as captured from mobile device GPS apps. Mobile device location data were collected by select apps and subsequently aggregated from about 15 million anonymous users who opted in to data collection for research purposes through a GDPR and CCPA compliant framework. The data are described more fully by Garber et al. (39).

Briefly, the dataset identifies unique mobile devices, the likely residential census block group for the device, and a series of coordinates for places the device visited over the course of a 1-month period in 2017. For each device, we assigned a quantile of the median household income and the proportion of the population who were Black, based on the value of the block group designated as the residential location for that device. Then we subsequently calculated average inequality indices in each census block group in Atlanta, based on the diversity or mixing of unique devices during travel away from their place of residence (40). The result is a set of two “inequality” indices indicating the average racial or economic diversity in a place based on daily activity, and not just based on residents.

Linear probability models were used to estimate prevalence differences (PD) for the associations of maternal level of education, health insurance type, recruitment hospital, and residential neighborhood socio-contextual factors with vaginal microbiome CST, and joint effects were also evaluated. We decided *a priori* to evaluate joint effects using absolute rather than relative models, so that we could interpret departures from additivity. We selected linear probability models because they provided estimates on the additive scale and yielded very similar results to the binomial identity models, which would not consistently converge across variable combinations. The outcome of interest, vaginal microbiome composition, was dichotomized into two groups: *Lactobacillus iners-*dominant and diverse community state types (CST III and IV), and *Lactobacillus* (non-*iners*)—dominant community state types (CST I, II, and V) based on the association of these community state types with an important pregnancy outcome, preterm birth in this same cohort (8). For the remainder of the paper, the former will be referred to as *L. iners*/diverse, and the latter will be referred to as Lacto-dominant. Due to the high prevalence of *L. iners*/diverse among the participants in this study, we chose to model the probabilities of women being in the Lacto-dominant group, so as to have a less common outcome.

We evaluated sensitivity of our findings to assumptions of independence by fitting GEE models clustering women by census tract, and we also estimated polytomous rather than binary outcome models to assess whether disaggregating the outcome from two groups to separate CST groups altered results. In both cases, analyses yielded similar results, but with substantially lower precision in polytomous models owing to smaller class-specific sample sizes; we therefore chose to present simpler binary model results.

**Figure 1:**
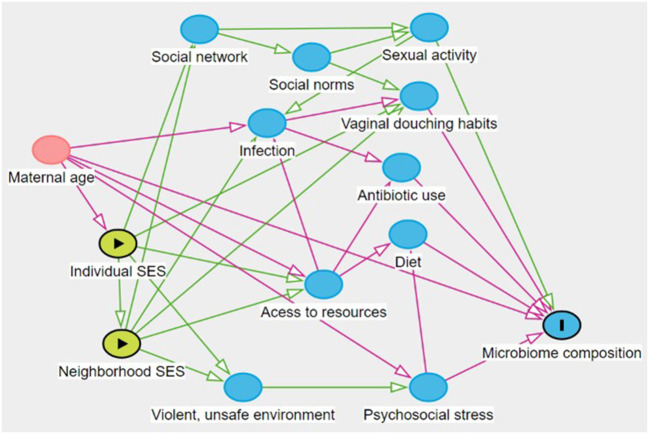
Hypothesized causal structure. This figure created at http://www.dagitty.net/ (41).

Based on literature review and DAG construction (see Figure 1), we acknowledge that many covariates are plausibly predictive of vaginal microbiome community state type, including douching, antibiotics, and sexual activity. We hypothesize that age is associated with individual and area-based socioeconomic status, with life experiences and behaviors, and with vaginal microbiome, and thus we adjust for participant age as a plausible confounder. However, we hypothesize that the other covariates are not causes of individual SES, but rather can be viewed as causal descendants or consequents of individual or area-based SES. As such, they are mediators, and not adjusted in our models.

To distinguish the effects of local racial and income diversity from the residential values, models including local income homogeneity are additionally adjusted for residential median household income, and the local racial homogeneity models are additionally adjusted for residential percentage Black population. Median household income and percentage Black population variables were obtained from the American Community Survey. After checking to see that the neighborhood variables were approximately normally distributed, we decided to calculate prevalence differences in the dependent variable contrasting women experiencing one standard deviation above the mean of a given predictor to those experiencing one standard deviation below the mean. Since we were using the common referent approach for the evaluation of joint effects, the reference group for each interaction was selected such that prevalence differences were for the most part positive (exception in **Table 4**). In other words, the common referent was selected as the exposure combination with the lowest predicted prevalence of Lacto-dominant community state types in the modeled interactions.

## Results

Among the 439 women included in this study, 368 (83.8%) had *L. iners*/diverse community state types, and about 53.8% of all women had a previous birth. Maternal education level, maternal insurance type, and maternal recruitment hospital were all notably different between the Lacto-dominant and *L. iners*/diverse groups. For example, 45.1% of the women in the Lacto-dominant group were college graduates, compared to only 10.9% in the *L. iners*/diverse group. Likewise, while 42.3% of women in the Lacto-dominant group had private insurance, only 17.7% had private insurance in the *L. iners*/diverse group. Residential neighborhood factors seemed more similar between the two groups, though neighborhood deprivation index and violent crime rate were noticeably higher in the *L. iners*/diverse group, and census response rate was noticeably higher in the Lacto-dominant group. There was an age difference between the two groups, and due to our hypothesized causal structure, we adjusted for age in all of our models (Table 1).

**Table 1:** Socio-demographic characteristics of study participants by vaginal microbiome community state type assignment.

	**All community state types**	***Lactobacillus* (non-*iners*) dominant^a^**	***Lactobacillus iners* dominant or diverse^b^**	***p*-Value**

	**(*****N*** = **439)**	**(*****N*** = **71)**	**(*****N*** = **368)**	
Age in years [mean (SD)]	24.9 (4.8)	26.4 (5.4)	24.6 (4.7)	0.004
**Education level (%)**				
Less than high school	67 (15.3)	6 (8.5)	61 (16.6)	<0.001
High school degree or GED	173 (39.4)	18 (25.4)	155 (42.1)	
Some college	127 (28.9)	15 (21.1)	112 (30.4)	
College graduate	72 (16.4)	32 (45.1)	40 (10.9)	
**Insurance during pregnancy (%)**				
Low income Medicaid	161 (36.7)	14 (19.7)	147 (39.9)	<0.001
RSM Medicaid (Medicaid during pregnancy)	183 (41.7)	27 (38.0)	156 (42.4)	
Private	95 (21.6)	30 (42.3)	65 (17.7)	
**Hospital (%)**				
Public	262 (59.7)	26 (36.6)	236 (64.1)	<0.001
Private	177 (40.3)	45 (63.4)	132 (35.9)	
Prior birth (%)	236 (53.8)	36 (50.7)	200 (54.3)	0.664
Census response rate [mean (SD)]	58.5 (10.6)	61.1 (10.5)	58.0 (10.6)	0.023
Neighborhood deprivation index [mean (SD)]	0.6 (0.3)	0.5 (0.2)	0.6 (0.3)	0.030
Violent crime rate [mean (SD)]	14.8 (12.9)	12.2 (11.6)	15.4 (13.1)	0.061
Percentage of households that moved in the past 12 months [mean (SD)]	0.2 (0.1)	0.2 (0.1)	0.2 (0.1)	0.126
Local income homogeneity [mean (SD)]	0.4 (0.2)	0.4 (0.2)	0.4 (0.2)	0.849
Local racial homogeneity [mean (SD)]	0.7 (0.3)	0.7 (0.3)	0.7 (0.3)	0.648

^a^Community State Types I, II, and V.

^b^Community State Types III and IV.

With regards to the analysis of the joint effects of individual socioeconomic status indicators with residential neighborhood factors on the prevalence of Lacto-dominant vaginal microbiome composition, we saw evidence of departure from additivity in almost all of our models, although not all *p*-values for the interaction terms were significant at alpha = 0.05. In Table 2, when looking at the joint effects of neighborhood census response rate and maternal education level, we see that, compared to non-college graduates who live in neighborhoods where the census response rate is one standard deviation below the mean, (a) non-college graduates who live in neighborhoods where the census response rate is one standard deviation above the mean have a 0.3% higher prevalence of Lacto-dominant vaginal microbiome composition, (b) college graduates who live in neighborhoods where the census response rate is one standard deviation below the mean have a 21.8% higher prevalence of Lacto-dominant vaginal microbiome composition, and (c) college graduates who live in neighborhoods where the census response rate is one standard deviation above the mean (the doubly exposed) have a 41.7% higher prevalence of Lacto-dominant vaginal microbiome composition (*p*-value for interaction 0.066). The magnitude of joint effects suggests similarly strong departure from additivity for other interactions in Table 2, although with much less precision, and non-significant *p*-values. However, for local income homogeneity, the doubly exposed did not have the highest prevalence difference compared to the common referent, and it appears there could potentially be crossover interaction.

**Table 2:** Joint effects of maternal education and residential neighborhood factors on prevalence of a *Lactobacillus* (non-*iners*)—dominant vaginal microbiome.

**Residential neighborhood factors^a^**	**Prevalence differences for non-college graduates**	**Prevalence differences for college graduates**	***p*-Value for interaction term**
Below-average census response rate	Common referrent	***0.218 (0.019, 0.418)***	0.066
Above-average census response rate	0.003 (−0.068, 0.074)	***0.417 (0.253, 0.581)***	
Above-average neighborhood deprivation index	Common referrent	***0.312 (0.188, 0.435)***	0.466
Below-average neighborhood deprivation index	0.002 (−0.065, 0.069)	***0.380 (0.248, 0.512)***	
Above-average violent crime rate	Common referrent	***0.311 (0.147, 0.475)***	0.409
Below-average violent crime rate	0.017 (−0.056, 0.090)	***0.413 (0.290, 0.537)***	
Above-average percentage of households that moved	Common referrent	***0.301 (0.155, 0.448)***	0.504
Below-average percentage of households that moved	0.034 (−0.041, 0.108)	***0.402 (0.273, 0.532)***	
Below-average local income homogeneity^b^	Common referrent	***0.390 (0.271, 0.509)***	0.103
Above-average local income homegeneity^b^	0.054 (−0.020, 0.127)	***0.302 (0.162, 0.443)***	
Below-average local racial homogeneity^c^	Common referrent	***0.346 (0.225, 0.466)***	0.920
Above-average local racial homogeneity^c^	0.064 (−0.025, 0.153)	***0.401 (0.256, 0.546)***	

Results calculated using linear probability models and all models adjusted for age.

^a^Comparison of one standard deviation above the mean to one standard deviation below the mean.

^b^Additionally adjusted for residential median household income.

^c^Additionally adjusted for residential percentage Black population.

Bold italics indicate statistically significant results (at an alpha level of 0.05).

In Table 3, when looking at the joint effects of neighborhood deprivation index and maternal insurance type, we see that, compared to women without private insurance who live in neighborhoods where the neighborhood deprivation index is one standard deviation above the mean, (a) women without private insurance who live in neighborhoods where the neighborhood deprivation index is one standard deviation below the mean have a 1.1% higher prevalence of Lacto-dominant vaginal microbiome composition, (b) women with private insurance who live in neighborhoods where the neighborhood deprivation index is one standard deviation above the mean have a 12.4% higher prevalence of Lacto-dominant vaginal microbiome composition, and (c) women with private insurance who live in neighborhoods where the neighborhood deprivation index is one standard deviation below the mean (the doubly exposed) have a 24.5% higher prevalence of Lacto-dominant vaginal microbiome composition (*p*-value for interaction 0.085). The magnitude of joint effects estimates suggests more than additive effect among the doubly exposed, and similar evidence appears for the top three variable combinations in Table 3 albeit with less precision and non-significant *p*-values. However, for neighborhood percentage of households that moved, the effect among the doubly exposed appears to be less than additive. Additionally, for local income homogeneity and local racial homogeneity, the doubly exposed did not have the highest prevalence difference compared to the common referent, and crossover interaction seems like a possibility.

**Table 3:** Joint effects of maternal insurance type and residential neighborhood factors on prevalence of a *Lactobacillus* (non-*iners*)—dominant vaginal microbiome.

**Residential neighborhood factors^a^**	**Prevalence differences for women without private insurance**	**Prevalence differences for women with private insurance**	***p*-Value for interaction term**
Below-average census response rate	Common referrent	0.064 (−0.080, 0.207)	0.085
Above-average census response rate	0.012 (−0.073, 0.098)	***0.253 (0.138, 0.368)***	
Above-average neighborhood deprivation index	Common referrent	***0.124 (0.011, 0.237)***	0.240
Below-average neighborhood deprivation index	0.011 (−0.060, 0.081)	***0.245 (0.116, 0.373)***	
Above-average violent crime rate	Common referrent	0.135 (−0.041, 0.311)	0.518
Below-average violent crime rate	0.023 (−0.054, 0.101)	***0.231 (0.119, 0.343)***	
Above-average percentage of households that moved	Common referrent	***0.195 (0.061, 0.328)***	0.636
Below-average percentage of households that moved	0.054 (−0.026, 0.134)	***0.206 (0.091, 0.320)***	
Below-average local income homogeneity^b^	Common referrent	***0.253 (0.144, 0.362)***	***0.006***
Above-average local income homegeneity^b^	***0.082 (0.001, 0.163)***	***0.124 (0.004, 0.244)***	
Below-average local racial homogeneity^c^	Common referrent	***0.252 (0.141, 0.362)***	***0.021***
Above-average local racial homogeneity^c^	***0.112 (0.018, 0.206)***	***0.180 (0.049, 0.311)***	

Results calculated using linear probability models and all models adjusted for age.

^a^Comparison of one standard deviation above the mean to one standard deviation below the mean.

^b^Additionally adjusted for residential median household income.

^c^Additionally adjusted for residential percentage Black population.

Bold italics indicate statistically significant results (at an alpha level of 0.05).

**Figure 2:**
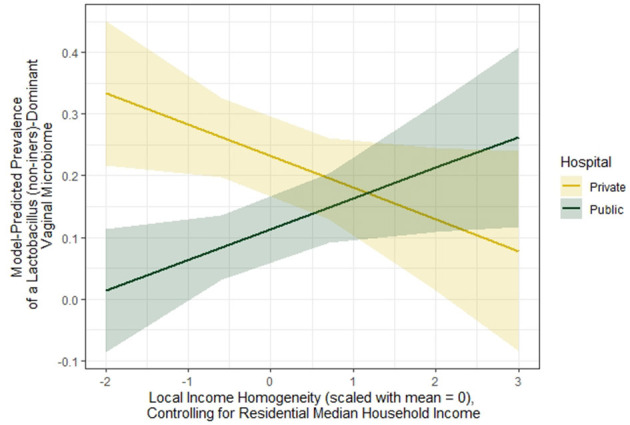
Illustrative interaction of crossover effect.

In Table 4, when looking at the joint effects of local income homogeneity (controlling for residential median household income) and maternal recruitment hospital, we see that, compared to women at the public hospital who live in neighborhoods where the local income homogeneity is one standard deviation below the mean (i.e. more economically diverse/heterogeneous), (a) women at the public hospital who live in neighborhoods where the local income homogeneity is one standard deviation above the mean (i.e. less economically diverse/heterogeneous) have a 10% higher prevalence of Lacto-dominant vaginal microbiome composition, (b) women at the private hospital who live in neighborhoods where the local income homogeneity is one standard deviation below the mean have a 21.9% higher prevalence of Lacto-dominant vaginal microbiome composition, and (c) women at the private hospital who live in neighborhoods where the local income homogeneity is one standard deviation above the mean (the doubly exposed) have a 11.7% higher prevalence of Lacto-dominant vaginal microbiome composition. The *p*-value for the interaction term is 0.004, and we can see a crossover effect clearly in Figure 2. Aside from local income homogeneity and local racial homogeneity, there appears to be strong evidence for a more than additive effect among the doubly exposed for the other variable combinations in Table 4.

**Table 4:** Joint effects of maternal recruitment hospital and residential neighborhood factors on prevalence of a *Lactobacillus* (non-*iners*)—dominant vaginal microbiome.

**Residential neighborhood factors^a^**	**Prevalence differences for women at the public hospital**	**Prevalence differences for women at the private hospital**	***p*-Value for interaction term**
Below-average census response rate	Common referrent	0.053 (-0.050, 0.156)	***0.048***
Above-average census response rate	−0.018 (−0.118, 0.082)	***0.196 (0.100, 0.293)***	
Above-average neighborhood deprivation index	Common referrent	***0.109 (0.029, 0.189)***	0.196
Below-average neighborhood deprivation index	−0.005 (−0.088, 0.078)	***0.193 (0.092, 0.294)***	
Above-average violent crime rate	Common referrent	***0.134 (0.018, 0.250)***	0.717
Below-average violent crime rate	0.021 (−0.068, 0.111)	***0.184 (0.091, 0.277)***	
Above-average percentage of households that moved	Common referrent	0.094 (-0.013, 0.201)	0.358
Below-average percentage of households that moved	0.018 (−0.073, 0.109)	***0.182 (0.085, 0.279)***	
Below-average local income homogeneity^b^	Common referrent	***0.219 (0.120, 0.319)***	***0.004***
Above-average local income homegeneity^b^	***0.100 (0.008, 0.191)***	***0.117 (0.018, 0.215)***	
Below-average local racial homogeneity^c^	Common referrent	***0.201 (0.099, 0.303)***	***0.042***
Above-average local racial homogeneity^c^	***0.113 (0.011, 0.216)***	***0.172 (0.064, 0.280)***	

Results calculated using linear probability models and all models adjusted for age.

^a^Comparison of one standard deviation above the mean to one standard deviation below the mean.

^b^Additionally adjusted for residential median household income.

^c^Additionally adjusted for residential percentage Black population.

Bold italics indicate statistically significant results (at an alpha level of 0.05).

## Discussion

When considering the joint effects of individual socioeconomic status indicators and residential neighborhood factors on vaginal microbiome composition, most combinations showed a greater than additive effect among the doubly exposed, although many interactions were imprecise and not statistically significant. In the case of local income homogeneity and local racial homogeneity, there appeared to be evidence of a crossover effect between those with less-advantaged individual socioeconomic status and those with more-advantaged individual socioeconomic status. The broad implications of these findings suggest that individual and area-based measures of SES may work synergistically in their influence of vaginal microbiome communities in pregnant African American women in this study.

There were two broad patterns evident in the joint effects estimations: evidence of supra-additive effects where the doubly-exposed experienced greater than the sum of the main effect exposures; and sub-additive effects where the doubly exposed had less risk than expected, consistent with the crossover pattern in Figure 2. In general, the supra-additive effects were evident in interactions between individual SES and NDI, census response rate, percentage of households that moved in the last year, and violent crime rate. For all of these variables, more (or less) is conceptually associated with concentrated advantage or disadvantage.

On the other hand, the sub-additive interactions generally occurred with the two local diversity measures. These are novel measures in that they capture a proxy for the general social mixing of people in each respondent's neighborhood through daytime activity. These measures are also notable because they draw attention to where populations are more homogenous or more heterogeneous. It would seem that among those with higher individual socioeconomic status, local income diversity/heterogeneity and local racial diversity/heterogeneity confer the most benefit; whereas, among those with lower individual socioeconomic status, local income homogeneity and local racial homogeneity confer the most benefit. The idea that neighborhood context can affect microbiome composition is a relatively new one, so these findings are very novel to our knowledge, but they build on related studies that have found relationships between psychosocial stress levels and bacterial vaginosis (17–21), as well as with other studies that have shown the effects of neighborhood context on other health outcomes (42).

In this study, we hypothesized that neighborhood material capital (proxied by the neighborhood deprivation index) might affect a woman's access to resources, which could influence her diet and substance-use behavior, among other things (24). Similarly, we thought that neighborhood social capital (indicated by census response rate) and residential stability (proxied by the percentage of households that moved in the past year) could impact a woman's social support, norms and beliefs around hygiene practices, and sexual activity (25–27). We additionally hypothesized that neighborhood measures of stressful or violent environments (indicated by violent crime rate) might be correlated with a woman's psychosocial health, and that neighborhood measures of inequality and diversity might correspond to social hierarchy and power structures, which could affect both access to resources and individual stress levels (28, 29). All of these factors could theoretically in turn contribute to vaginal microbiome composition. Main effects age-adjusted models analyzing these contextual factors provided evidence that neighborhood deprivation, violent crime rate, and census response rate were independently associated with vaginal microbiome composition, potentially through the pathways hypothesized above. It will be important for future studies to continue looking into the relationships explored in this study, especially among study populations with larger sample sizes and thus greater power to detect meaningful interactions.

There are several clinical and public health implications from these findings. First of all, these findings provide evidence in support of the ecosocial theoretical framework's embodiment theory (23, 43). The microbiome may be one important pathway for better understanding how social status and position, social experience, and broader structural social stratification can become biologically embodied.

Ecosocial theory also places importance on accountability and agency, especially with regards to structural factors (44). While the contextual social determinants we examined in this study were more proximate than structural, it is notable that despite the substantial individual socioeconomic diversity within this sample of Black pregnant women, there are relatively smaller differences in neighborhood factors. This observation may reflect the importance of the structural factors that shape the range of environments even in a city like Atlanta.

Additionally, for pregnant women who may be at higher risk for vaginal microbiome compositions associated with worse health outcomes, there may be additional resources and support that can be provided to them in the course of their prenatal care, especially if they live in neighborhoods with a lack of resource concentration.

Several limitations were present in our study. First of all, we had small sample size for estimating joint effects, and as a result had imprecise effect estimates. Consequently, these findings should be taken as exploratory and need to be replicated in larger samples. A second limitation was in our use of the local income homogeneity and local racial homogeneity measures. These were imperfect in that, although they used mobile phone movement to approximate these inequalities, they tagged each phone not with the actual user's race and income, but rather with the average race and income in the neighborhood where the mobile phone user lived. There is therefore potential for misclassification in these measures. Additionally, our interest is in the social epidemiology of a clinically meaningful health state as summarized by our findings comparing non-*iners* Lacto-dominant CST to *L. iners* dominant or diverse CST. However, categorizing the outcome dichotomously could have obscured differences between the CSTs that we grouped together.

In conclusion, it seems that in this study, aspects of residential neighborhood context were experienced differently by women on the basis of their individual resources, and that the joint effects of these exposures on vaginal microbiome composition showed a departure from simple additivity. These findings highlight the importance of considering both individual and neighborhood factors when evaluating risk for health outcomes.

## Data availability statement

The datasets presented in this study can be found in online repositories. The names of the repository and accession number can be found below: https://www.ncbi.nlm.nih.gov/bioproject, PRJNA725416.

## Ethics statement

The studies involving human participants were reviewed and approved by Emory University IRB. The patients/participants provided their written informed consent to participate in this study.

## Author contributions

MD, AD, EC, and MK contributed to conception and design of the study. MD and MK performed the statistical analysis. MD wrote the first draft of the manuscript. All authors contributed to manuscript revision, read, and approved the submitted version.
