# Corrigendum: Joint effects of individual socioeconomic status and residential neighborhood context on vaginal microbiome composition

**DOI:** 10.3389/fpubh.2023.1192743

**Published:** 2023-05-10

**Authors:** Meredith Dixon, Anne L. Dunlop, Elizabeth J. Corwin, Michael R. Kramer

**Affiliations:** ^1^Department of Epidemiology, Rollins School of Public Health, Emory University, Atlanta, GA, United States; ^2^Department of Gynecology and Obstetrics, Emory University School of Medicine, Atlanta, GA, United States; ^3^Columbia University School of Nursing, New York, NY, United States

**Keywords:** microbiota, social environment, neighborhood characteristics, pregnancy, United States, dysbiosis

## Text correction

In the published article, there was an error.

A correction has been made to Methods section, Paragraph 5. This section previously stated:

“To quantify aspects of the racial and economic diversity of each woman's neighborhood during the day (e.g., from non-residents visiting), we used a large database of daily mobility as captured from mobile phone GPS apps, as provided by Cuebiq, a data aggregation firm. Cuebiq aggregated mobile phone location data, which are collected by select smartphone apps from about 15 million anonymous users who opted in to data collection for research purposes through a GDPR and CCPA compliant framework.”

The corrected sentence appears below:

“To quantify aspects of the racial and economic diversity of each woman's neighborhood during the day (e.g., from non-residents visiting), we used a large database of daily mobility as captured from mobile device GPS apps. Mobile device location data were collected by select apps and subsequently aggregated from about 15 million anonymous users who opted in to data collection for research purposes through a GDPR and CCPA compliant framework.”

The authors apologize for this error and state that this does not change the scientific conclusions of the article in any way. The original article has been updated.

